# Post-transplant lymphoproliferative disorders and Epstein-Barr virus DNAemia in a cohort of lung transplant recipients

**DOI:** 10.1186/1743-422X-8-421

**Published:** 2011-09-05

**Authors:** Fausto Baldanti, Vanina Rognoni, Alessandro Cascina, Tiberio Oggionni, Carmine Tinelli, Federica Meloni

**Affiliations:** 1 Laboratori Sperimentali di Ricerca, Fondazione IRCCS Policlinico San Matteo, 27100 Pavia, Italy; 2 Clinica di Malattie dell'Apparato Respiratorio, Fondazione IRCCS Policlinico San Matteo, 27100 Pavia, Italy; 3 Servizio di Epidemiologia Clinica e Biometria, Fondazione IRCCS Policlinico San Matteo, 27100 Pavia, Italy

**Keywords:** EBV, PTLD, DNAemia, lung transplant recipients

## Abstract

**Background:**

Post-transplant lymphoproliferative disorders (PTLD) are serious complications in lung transplant recipients. No consensus on EBV DNAemia levels predictive of PTLD has been reached. In addition, in many instances EBV DNAemia is determined in patients with suggestive symptoms only.

**Methods:**

The characteristics of five patients with PTLD as well as the prevalence of EBV DNAmia in a cohort of 137 consecutive patients receiving lung transplantation are described.

**Results:**

Twenty-six out of 137 patients (18.9%) were excluded from the analysis because lost at follow-up or dead from PTLD-independent reasons within three months of transplantation. EBV DNA in peripheral blood mononuclear cells (PBMC) was determined in 83/111 patients (74.8%) because of potential PTLD-related symptoms, while 28 patients (25.2%) showed no symptoms and were not examined. EBV DNAemia was positive in 53/83 patients (63.8%), and negative in 30/83 patients (36.2%). PTLD was diagnosed in five (4.5%) patients at a median time of 270 (range 120-870) days following transplantation. All five PTLD (three large B-cell lymphomas, one Hodgkin lymphoma and one possible pre-neoplastic lesion) were potentially associated with EBV infection. However, only 3/5 patients with PTLD had detectable EBV DNAemia: < 1,000 copies EBV DNA/1 × 10^5 ^PBMC in one patient and > 1,000 copies EBV DNA/1 × 10^5 ^PBMC in two patients.

**Conclusion:**

A systematic multidisciplinary (clinical, radiologic, virologic and histologic) approach is mandatory for the diagnosis and management of PTLD in lung transplant recipients, while monitoring of symptomatic patients only may provide an incomplete or late picture of the clinical problem. In addition, staining for EBV antigens and quantification of EBV DNA in biopsy specimens should always be performed to understand the role of EBV infection in the pathogenesis of PTLD.

## Background

Post-transplant lymphoproliferative disorders (PTLD) represent serious infectious complications in lung transplant recipients, who are at a greater risk than kidney, heart and liver transplant recipients [1-3]. However, the heterogeneous spectrum of clinical conditions included in PTLD definition (ranging from polymorphic lymphoproliferation to aggressive lymphomas) [3,4], the wide time span from transplantation to emergence [2] and the debated pathogenetic role of Epstein-Barr virus (EBV) [2,4-6] make it difficult to perform cohort studies in transplant recipients [1-8]. In particular, the wide time interval to PTLD onset in different patient groups affects the feasibility of a tight monitoring of EBV DNA in blood, particularly in patients with late onset of PTLD, such as solid organ transplant recipients. Thus, systematic monitoring of EBV DNAemia in many transplant centers is often impossible as it depends on clinical manifestations suggestive of PTLD. For these reasons, the role of detection and quantification of EBV DNA in blood compartments (EBV DNAemia), used for monitoring patients at risk for PTLD [9-13] and guiding preemptive treatment [9,14-16] is still debated.

Here we describe the characteristics of five patients who developed PTLD and the prevalence of EBV DNAemia in peripheral blood mononuclear cells (PBMC) in a cohort of 137 consecutive patients submitted to lung transplantation in a single transplantation center in Northern Italy from 2000-2007. Diagnosis and treatment of this elusive disease remains a clinical challenge.

## Materials and methods

This retrospective study aimed at evaluating the prevalence and levels of EBV DNAemia in PBMC in a cohort of patients submitted to single-lung, double-lung or heart-lung transplantation in a single transplantation center in Northern Italy from 2000 to 2007.

From 2000 to 2003, EBV DNAemia was determined using a quantitative PCR technique [9], while from 2004 to 2007 a real-time PCR technique was adopted [17]. The two assays showed comparable sensitivity, being both able to reproducibly detect 10 EBV DNA copies in a background of 1 × 10^5 ^PBMC. In addition, the comparative analysis of a subset of PBMC samples as well as the results of an international quality control program QCMD http://www.qcmd.org showed agreement between the two PCR assays for quantification of EBV DNA levels (data not shown). Results were expressed as EBV DNA copy number/1 × 10^5 ^PBMC, and samples with no PCR signals were scored as containing < 10 EBV DNA copies/1 × 10^5 ^PBMC.

EBV DNAemia was prospectively determined when the patient exhibited symptoms or signs potentially associated with PTLD: fever of unknown origin, lymphoadenopathy, cytopenia, leukopenia, weight loss and asthenia. A presumptive diagnosis of PTLD was made based on virologic and radiologic findings and a definitive diagnosis was made by histologic or cytologic examination of tissue biopsies or needle aspirates.

Immune suppression therapy was reduced in patients showing EBV DNA values > 1,000 copies/1 × 10^5 ^PBMC [9]. Patients presenting with overt lymphomas were submitted to standard treatment protocols.

All the patients signed an informed consent at the time of transplantation. The study was approved by the Internal Review Board (protocol no. P-20080013903, Jun 3, 2008). Due to the retrospective nature of the study and the impossibility to obtain informed consent from patients deceased and lost at follow-up, the IRB allowed the analysis of anonymized stored samples and data (IRB protocol no. P-20020001513, Jan 18, 2010).

The Shapiro-Wilk's test was used to test the normal distribution of quantitative variables. If they were normally distributed, mean and standard deviation (SD) were used to summarize the results. Otherwise, median and Interquartile range (IQR; 25° - 75° percentile) were used. Specificity and sensitivity (with 95% Confidence Intervals) were used to compare positive and negative results. Differences between median EBV DNAemia levels at first detection and at peak of infection were evaluated with the Wilcoxon signed-rank test. The chi-squared statistics or Fisher's exact test, as appropriate, were applied to compare qualitative variables. P < 0.05 was considered statistically significant. All tests were two-sided. Data analysis was performed with the STATA statistical package (vers: 9; Stata Corporation, College Station, 2008, Texas, USA).

## Results

Stored data of 137 patients receiving a single-lung (n = 58), double-lung (n = 74) or heart-lung (n = 5) transplantation from January 2000 to June 2007 were retrospectively analyzed. Data have been prospectively generated and archived as part of routine monitoring of infectious complications in solid organs transplant recipients. Ninety-nine patients (72.2%) were male, median age at transplantation was 52 years (range 13-71).

Twenty-six out of 137 patients (18.9%) were excluded from the analysis because lost at follow-up or dead from PTLD-independent reasons within 3 months after transplantation. The remaining 111 patients (81.1%) were considered representative of the entire cohort, since no differences in sex, age at transplantation, type of transplantation and distribution of underlying diseases were observed (p > 0.05 for all parameters).

Twenty-eight out of 111 patients (25.2%) never showed symptoms suggestive of PTLD and were not examined for EBV DNAemia, while 83 (74.8%) patients were examined at least once (median test no. 3; IQR 2 - 5) due to the presence of potential PTLD-related symptoms.

EBV DNAemia was positive in 53/83 patients (63.8%), while 30/83 patients (36.2%) were EBV DNA-negative. EBV DNAemia was first detected at a median time of 263 days from transplantation (IQR: 80 - 952). Among the 53 EBV DNA-positive patients, 10 (18.8%) showed EBV DNAemia levels > 1,000 copies/1 × 10^5 ^PBMC (median 2,430; range 1,208-1,311,180), while 43 patients showed lower values (median, 116; range 10-704).

Overall, PTLD was diagnosed in 5/111 (4.5%) patients at a median time of 270 days after transplantation (range 120-870) in the presence of different EBV DNA levels in PBMC: 2 patients scored as EBV DNAemia negative, 1 patient showed < 1,000 copies EBV DNA/1 × 10^5 ^PBMC and 2 patients showed > 1,000 copies EBV DNA/1 × 10^5 ^PBMC. Characteristics of the five patients with PTLD are summarized in Table 1.

**Table 1:** Characteristics of 5 lung transplant recipients patients with posttransplant lymphoproliferative disorder (PTLD).

Pt. #, age, sex	Underlying disease	Date of TX	Type of TX	Onset of PTLD	Immuno suppressive treatment (dosage)	PTLD description	EBV DNA copies/10^5^PBMC
1, 29, M	cystic fibrosis	Dec. 2000	double-lung	May 2003	Ciclosporin (350 mg/die) Steroid (5 mg/die)	Laterocervical HL	1,760
2, 25, F	cystic fibrosis	Sep. 2001	double-lung	May 2002	Ciclosporin (150 mg/die) Steroid (40 mg/die)	Mediastinic large B-cells NHL	< 10
3, 53, M	sarcoidosis	Dec. 2001	single-lung	Nov. 2002	Ciclosporin (250 mg/die) Steroid (25 mg/die)	Mediastinic large B-cells NHL	< 10
4, 39, M	Istiocytosis X	Dec. 2004	double-lung	May 2005	Ciclosporin (550 mg/die) Steroid (5 mg/die)	thoracic localization of EBV-positive B-cell lymphoma.	307
5, 63, M	Idiopathic pulmonary fibrosis	Aug. 2006	double-lung	Dec. 2006	Ciclosporin (300 mg/die) Steroid (20 mg/die) Mycophenolate mofetil (1000 mg/die)	Single nodule in lower left lobe, double mediastinic nodule (at histology, no tumor markers) 751,000 EBV DNA copies/μg tissue DNA, 8,500,000 copies/ml BAL	15, 289

TX: transplantation; PBMC: peripheral blood mononuclear cells; M: male; F: female; HL: Hodgkin lymphoma; NHL: non-Hodgkin lymphoma.

**Figure 1:**
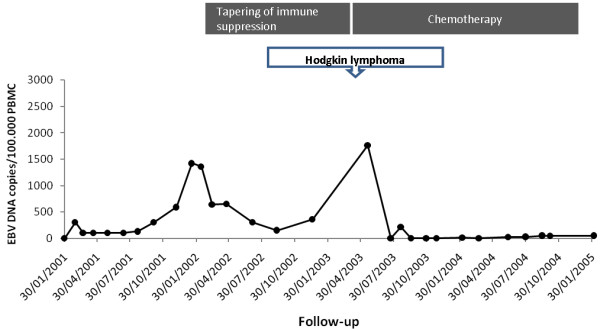
**Kinetics of EBV DNAemia in a lung transplant recipient developing primary EBV infection and PTLD in the post-transplant period**. The dotted line indicates EBV DNAemia levels. The dark-grey boxes indicate treatment periods. The time of PTLD diagnosis is indicated by the arrow.

In more detail, Patient #1, developed primary EBV infection two months after transplantation and low-level EBV DNAemia persisted for one year (Figure 1). Twenty-five months after transplantation, EBV DNAemia peaked at 1,420 copies/10^5 ^PBMC in the absence of PTLD symptoms. Tapering of immune suppression was associated with reduction of EBV DNAemia in PBMC (Figure 1). Three and a half years post transplantation the patient presented with laterocervical Hodgkin lymphoma (mixed cell) in the presence of 1,760 copies EBV DNA/1 × 10^5 ^PBMC. Following multiple treatment courses with interferon and anti-CD20 MAb, remission of the lymphoma was achieved. In parallel, disappearance of EBV DNA in blood was observed (Figure 1). This patient died five years and eight months after transplantation of bronchiolitis obliterans syndrome.

Patient #2 developed a broncho-mediastinic diffuse large B-cell lymphoma eight months after transplantation. EBV DNA in PBMC was negative. The disease was refractory to chemotherapy and the patient died eight months later.

Patient #3, developed a diffuse large B-cell lymphoma in the upper lobe of the transplanted lung associated with homolateral hilar-mediastinic lymphadenopathy 11 months after transplantation. EBV DNAemia was negative. No remission was achieved despite chemotherapy and the patient died three months later.

In patient #4, lung nodules suggestive of lymphoma were detected by computerized tomography (CT) four months after transplantation. Leukopenia (2,290 cells/μl) associated with marked lymphopenia (8%) was simultaneously recorded. EBV DNAemia was positive at a low level (183 copies/1 × 10^5 ^PBMC). One month later, fever and asthenia prompted a second CT scan analysis which revealed significant hilar bronchial and vascular infiltrate of the left lung. Bronchoscopy showed extrinsic occlusion of the major left bronchial lumen and concomitant infiltrate of the bronchial wall. Histology confirmed the presence of a diffuse large B-cell lymphoma. Immunohistochemistry documented the presence of EBV antigens in more than 80% of neoplastic cells. EBV DNAemia was still low (395 copies/1 × 10^5 ^PBMC). Leukopenia (3,560 cells/μl) and lymphopenia (8%) still persisted. Remission of the B-cell lymphoma was not achieved following seven courses of chemotherapy. Two years after transplantation, 19 copies EBV DNA/1 × 10^5 ^PBMC were shown in the presence of normal leukocyte counts. The patient died three months later of a massive hemophtysis.

**Figure 2:**
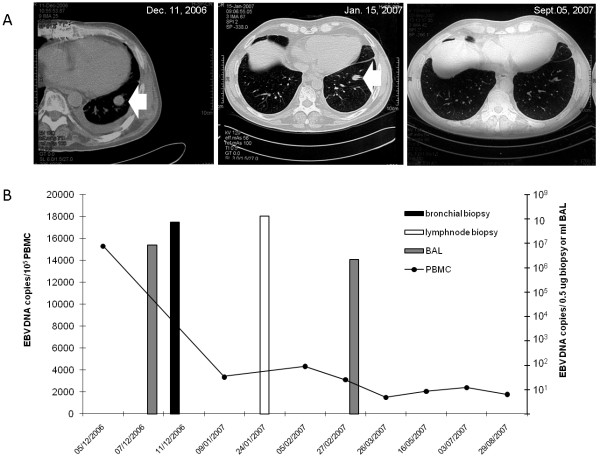
**Kinetics of EBV DNAemia and computerized tomography scan analysis in a patient with PTLD**. (A) Monitoring by computerized tomography scan analysis of an intrathoracic PTLD nodule (white arrow) in a lung transplant recipient. (B) EBV DNA levels in peripheral blood mononuclear cells (PBMC), bronchoalveolar lavage (BAL) and biopsy specimens.

In patient #5 a routine CT scan analysis performed four months after transplantation showed a non-infiltrative nodular lesion lacking contrast enhancement in the left lower lobe. Positron emission tomography (PET) analysis did not show increased glucose uptake in the lesion, while two additional controlateral mediastinal nodules showed increased glucose uptake. Histologic examination of the lung lesion showed the presence of nonspecific inflammatory infiltrates. PCR showed high EBV DNA levels in PBMC (15,289 copies/1 × 10^5 ^PBMC), lung nodule aspirate (751,000 copies/μg tissue DNA) and bronchoalveolar lavage (BAL) (8,500,000 copies/ml). Taken together these data were suggestive of a pre-neoplastic EBV-associated PTLD. The patient was submitted to significant tapering of the immune suppressive regimen. One month later, a reduction in the nodule size and EBV DNA level in PBMC (Figure 2) were observed. On the other hand, PET analysis showed the persistence of increased glucose uptake in mediastinic lesions. EBV DNA determination in a lymphnode biopsy showed 131,118,000 EBV DNA copies/0.5 ug tissue DNA (Figure 2). Two months later, EBV DNAemia levels were reduced by 4.9-fold (3,108 copies/1 × 10^5 ^PBMC) and EBV DNA levels in BAL by 3.8-fold (2,213,300 EBV DNA copies/ml BAL) (Figure 2). The size of the mediastinic nodules was reduced as well, and the intraparenchymal lesion disappeared (Figure 2). Nine months after transplantation, CT scan analysis showed the absence of lung and mediastinal nodules, while EBV DNAemia persisted at levels > 1,000 EBV DNA copies/1 × 10^5 ^PBMC (Figure 2). The patient is currently alive and well.

Tapering of immune suppression was associated with disappearance of EBV DNAemia in the other eight patients with EBV DNAemia levels > 1,000 copies in the absence of radiologic findings for PTLD.

## Discussion

PTLD had a prevalence of 4.5% in patients with > 3 months follow-up. This data is in agreement with previous reports [3,18]. Despite the low number of PTLD in our series, some conclusions can be drawn. Although all five confirmed PTLD (3 large B-cell lymphomas, 1 Hodgkin lymphoma and 1 possible pre-neoplastic lesion) were potentially associated with EBV infection [3], only 3 patients had detectable EBV DNAemia (patient #1, 4 and 5). Thus, the two patients with negative EBV DNAemia (patient #2 and 3) were either carrying a B-cell PTLD not driven by EBV [5] or the effect of EBV infection on lymphoproliferation had been localized at the intra-thoracic level [19]. Unfortunately, biopsies were not analyzed for EBV antigens or nucleic acids, and neither hypothesis can be dismissed. Among the three EBV DNAemia-positive patients with PTLD, two showed high and one low EBV DNAemia levels. However, EBV DNAemia could have been underestimated in the latter due to lymphopenia

In conclusion: i) a combined clinical, radiological, virological and histologic approach is mandatory for the diagnosis and management of PTLD, ii) staining for EBV antigens and quantification of EBV DNA in biopsy specimens should always be performed in parallel, iii) when critically evaluated in the clinical context, EBV DNAemia remains a useful parameter to follow-up patients at risk for PTLD, although the present data suggest that this disorder may occur also in the absence of detectable EBV DNAemia, iv) the role of other biologic factors remains to be elucidated.

## Competing interests

The authors declare that they have no competing interests.

## Authors' contributions

FB study design, data analysis, manuscript preparation and fund raising. VR, AC, TO data collection and analysis. CT statistical analysis. FM data analysis and manuscript preparation.

All authors read and approved the final manuscript.
